# The Neurophysiological Impact of Subacute Stroke: Changes in Cortical Oscillations Evoked by Bimanual Finger Movement

**DOI:** 10.1155/2022/9772147

**Published:** 2022-01-24

**Authors:** Ana Dionísio, Rita Gouveia, João Castelhano, Isabel Catarina Duarte, Gustavo C. Santo, João Sargento-Freitas, Miguel Castelo-Branco

**Affiliations:** ^1^Institute of Nuclear Sciences Applied to Health ICNAS, Coimbra Institute for Biomedical Imaging and Translational Research CIBIT, University of Coimbra, Coimbra, Portugal; ^2^Faculty of Sciences and Technology FCTUC, Department of Physics, University of Coimbra, Coimbra, Portugal; ^3^Faculty of Medicine FMUC, University of Coimbra, Coimbra, Portugal; ^4^Stroke Unit, Neurology Department, Coimbra Hospital and University Centre, Coimbra, Portugal; ^5^Brain Imaging Network, University of Coimbra, Coimbra, Portugal

## Abstract

**Introduction:**

To design more effective interventions, such as neurostimulation, for stroke rehabilitation, there is a need to understand early physiological changes that take place that may be relevant for clinical monitoring. We aimed to study changes in neurophysiology following *recent* ischemic stroke, both at rest and with motor planning and execution.

**Materials and Methods:**

We included 10 poststroke patients, between 7 and 10 days after stroke, and 20 age-matched controls to assess changes in cortical motor output via transcranial magnetic stimulation and in dynamics of oscillations, as recorded using electroencephalography (EEG).

**Results:**

We found significant differences in cortical oscillatory patterns comparing stroke patients with healthy participants, particularly in the beta rhythm during motor planning (*p* = 0.011) and execution (*p* = 0.004) of a complex movement with fingers from both hands simultaneously. *Discussion*. The stroke lesion induced a decrease in event-related desynchronization in patients, in comparison to controls, providing evidence for decreased disinhibition.

**Conclusions:**

After a stroke lesion, the dynamics of cortical oscillations is changed, with an increasing neural beta synchronization in the course of motor preparation and performance of complex bimanual finger tasks. The observed patterns may provide a potential functional measure that could be used to monitor and design interventional approaches in subacute stages.

## 1. Introduction

Stroke represents the third major cause of death and is one of the leading sources of disability, contributing to a decline in the global quality of life. Although several approaches are applied to the rehabilitation of patients, current interventions lack efficacy [1].

In order to develop new and more effective interventions for neurorehabilitation, and particularly, for the rehabilitation of stroke patients, it is fundamental to understand subacute physiological changes of potential neuroplastic significance following the event. After a brain lesion, neural networks are damaged, which triggers the reorganization of neural connectivity and brain rhythms. Plastic changes may occur not only on the lesioned but also in the contralateral hemisphere [2]. It is frequently reported in the literature that the activity of the unaffected hemisphere increases in the first days after the cerebrovascular accident [2, 3]. After this period, at 3 to 6 months following the event, a relative increase in the activity of the areas adjacent to the lesion is frequently observed, concurrent with functional improvements [3].

Functional techniques to assess brain changes include electroencephalography, magnetoencephalography, and functional magnetic resonance imaging [2]. Electroencephalography (EEG) can potentially contribute to the understanding of the physiology of brain reorganization [4], in particular in which concerns the study of dynamics of oscillations [5].

Brain oscillations can appear at diverse frequencies, associated to distinct levels of synchrony in neuronal networks [6]. The visual alpha rhythm is known to respond to a stimulus or instruction with a decrease in amplitude or power, resulting in an event-related desynchronization (ERD). Synchronization (ERS) occurs in the absence of stimuli or idle states. It is therefore believed that alpha ERS is associated to cortical inhibition, whereas ERD is related to the reduction of inhibition, in turn [7]. Current knowledge, nevertheless, also points out a role for other types of alpha rhythm in attention and conscious awareness [8].

Performing a voluntary movement or receiving instructions to execute a motor task are generally associated with a decrease in upper alpha (mu rhythm) and in beta rhythms [6, 7], in those regions around sensorimotor areas [6, 9]. This reduction of movement-related beta power is thought to be associated with the excitability of the primary motor cortex and to be affected by GABA (gamma-aminobutyric acid) levels [10].

Preparation and execution of motor tasks might reveal altered activity patterns in stroke, which may have significant implications for the design of therapeutic interventions [11]. Changes in neural synchronization and oscillatory activities can play a role in the pathophysiology of distinct disorders, such as in stroke [7]. The poststroke changes in brain oscillations, particularly those accompanying movements of the impaired limbs, are worthy of further research [10]. Therefore, exploration of biomarkers to strengthen stroke investigation has been advocated [12], and recent works have been studying EEG activity in stroke, along with motor tasks, such as unilateral [11–13] or bilateral wrist movements [13].

Here, we determined motor thresholds as a measure of cortical excitability and assessed ERD and ERS in the course of motor tasks, both in healthy subjects and in poststroke patients. To the best of our knowledge, this is the first time that the neurophysiology of stroke patients is analysed shortly after the event (between 7 and 10 days poststroke) by EEG preceding and during simple and complex fine-tuned unilateral and bilateral motor tasks performed with both the affected and unaffected arms and hands, and a direct comparison with a control healthy sample that did the same experiment is provided. Our aim was to study the impact of a subacute ischemic stroke in brain neurophysiology at rest and during motor preparation and execution. Moreover, we investigated whether significant changes in the EEG brain activity pattern following stroke could be correlated with the motor performance of the affected upper limb, assessed by the Wolf Motor Function Test.

## 2. Materials and Methods

The present work was conducted in accordance with the Declaration of Helsinki and received the approval from the Ethics Committee of the Faculty of Medicine of the University of Coimbra. Written informed consent was collected from each participant.

### 2.1. Sample

We included 10 patients who were recruited from the Neurology Department of the Coimbra University Hospital after a first-ever middle cerebral artery stroke and fulfilled our requirements: (i) 18 to 85 years of age; (ii) corticosubcortical ischemic lesion; (iii) stroke event 7 ± 3 days before; (iv) motor deficit of the upper extremity; (v) score ≤ 1 on the modified Rankin Scale, previous to the event; and (vi) ability to comprehend and follow the tasks. On the other hand, patients who (i) were not clinically stable, (ii) were diagnosed with cognitive impairment or dementia, (iii) had history of epileptic seizures, (iv) presented posterior or global aphasia, (v) presented neglect, (vi) abused drugs or alcohol, or (vii) presented contraindications to transcranial magnetic stimulation as assessed by a questionnaire based on published guidelines [14, 15] were excluded. Moreover, we recruited 20 age-matched healthy controls. Demographic data from the participants, both healthy individuals and stroke patients, is presented in Table 1.

In Table 2 we present some clinical data from our sample of stroke patients.

### 2.2. Wolf Motor Function Test (WMFT)

First of all, we have evaluated motor function of stroke patients by applying the WMFT. This test consisted on 15 timed tasks [16] that were performed with the affected upper limb. Each movement had a maximum length of 120 seconds. This way, if a patient could not perform the task, it was attributed a duration of 120 seconds. The quality of the movements was also evaluated by the functional ability scale (FAS) [17], wherein we attributed a score of “0” when a given movement was not performed and a maximum of “5” points per task, if it appeared to be normal, counting up to a maximum of 75 points.

### 2.3. Electroencephalography (EEG) Task

In this study, we have used the same methodological EEG procedure as used in our prior works addressing oscillatory changes induced by TMS [18, 19]. EEG was conducted using a SynAmps2 RT amplifier and Scan 4.5 software (Compumedics, Charlotte, NC). Electrodes' positioning was based on the International 10-20 montage, through the use of a 64-channel cap (QuickCap, NeuroScan, USA), including a ground placed in the forehead, close to FPZ, and online reference channel close to CZ. The signal was acquired at a 1000 Hz sampling rate. We applied a high-pass filter from the DC level and a low-pass at 200 Hz. For the study of posterior alpha rhythms, we recorded electrical activity during 180 seconds of eyes opening and closure task (blocks of 10 sec). To analyse differences in cortical oscillatory patterns along motor preparation and execution, we instructed participants to perform two different motor tasks, namely, 90° shoulder flexion and thumb opposition. Motor tasks were executed with both upper limbs, first individually and then simultaneously. Each participant was instructed to perform the movement and sustain it for 15 seconds and then reposition and rest for another 15 seconds. Subjects performed 6 trials of 30 seconds per movement, divided into blocks of 6 secs locked to the beginning of the task, in a 180 sec experiment for each movement, totalizing 540 secs per task and 1080 secs for the complete motor paradigm. Triggers time-locked to the beginning of each movement were inserted in the EEG file during the online recording of all tasks.

**Figure 1:**
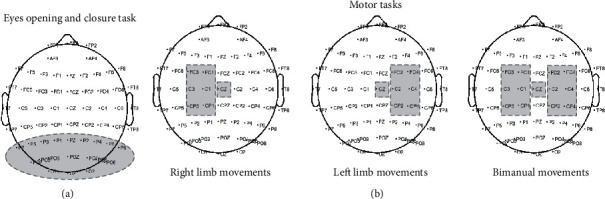
Schematic representation of the electrode clusters selected for the quantification of visual alpha (a) and mu and beta motor rhythms (b).

We carried out signal analysis with Scan 4.5 software (Compumedics, Charlotte, NC) and with the MATLAB (version R2017b, The MathWorks, USA) toolbox EEGLAB v.14.1.1b [20]. After recording data, we filtered the signal offline from 1 to 45 Hz and downsampled data to 250 Hz. The average of all channels was used for offline rereference. Moreover, we ran custom MATLAB scripts (adapted from our previous works by Castelhano et al. [21] and by Silva et al. [22]) to quantify alpha (8-13 Hz), mu (10-12 Hz), and beta (15-25 Hz) power, in the specified electrode clusters (Figure 1).

We selected posterior electrodes for the analysis of visual alpha in the occipital area. For the motor tasks, in order to quantify motor rhythms, namely, mu and beta bands, we selected those electrodes located on the central motor regions.

During the acquisition, we inserted online manual triggers in the EEG file marking the events that could disturb the signal and should be rejected. We have also used an offline procedure implemented in EEGLAB, with default parameters, that included a 1 Hz high-pass filtering step, a voltage threshold, and a visual confirmation of the muscle artifacts. Moreover, we computed Independent Component Analysis for further cleaning of the data and to remove components such as eye blinks. The pseudo-Wigner-Ville transformation was applied, according to the works by Uhlhaas et al. [23] and others [21, 24–26], for performing a time-frequency analysis. The amplitude and phase were computed for all periods of interest, with epochs being defined ahead, for all frequency bins from 5 to 40 Hz (resolution of 1 Hz/frequency bin). Posterior alpha rhythm was assessed from -2000 to 10000 milliseconds, where the period between -2000 milliseconds and 0 was defined as the baseline. Quantification of motor rhythms, in turn, was computed between -2000 and 0 milliseconds for premovement and preparation and from 0 until 4000 milliseconds, time-locked to the beginning of the movement. We also mapped topographical distribution in EEGLAB, using default parameters.

In addition, for patients, we determined beta power for one central electrode in each hemisphere to assess whether changes in relation to controls were central and bilateral or if they were due to hemispheric asymmetries induced by the lesion. This analysis was carried out over C3 and C4, where oscillations such as the mu rhythm are reported to show maximum amplitude [27]. One patient was not able to complete the EEG recording; therefore, for EEG analysis, we had a sample size of 9 patients and 20 healthy volunteers.

### 2.4. Transcranial Magnetic Stimulation (TMS)

We applied single pulses of transcranial magnetic stimulation to the unaffected primary motor cortex (M1) of patients and randomly to the right or left M1 of healthy subjects, at 45° to the sagittal plane, via a figure-of-eight coil plugged into a MagPro X100 magnetic stimulator (MagVenture, Denmark). Active motor threshold (aMT) was determined during isometric contraction of the upper limbs, being defined as the lowest intensity that elicited a minimal visible muscle twitch on the hand. The aMT was selected as a measure of cortical excitability, rather than the resting motor threshold (rMT), since it is reported that it presents less variability than rMT, due to the lower variability in the spinal excitability, associated with muscle contraction [28].

### 2.5. Statistics

Statistical tests were computed on the SPSS Statistics software, version 24 (IBM SPSS Statistics, IBM Corporation, Chicago, IL), and we adopted a significance level of 5% for all tests. We ran Mann–Whitney *U* test to address differences between healthy individuals and stroke patients, in cortical excitability and oscillatory patterns, comparing groups regarding active motor threshold, alpha power (8-13 Hz), and the ERD in mu (10-12 Hz) and beta (15-25 Hz) rhythms. Moreover, we applied the same test to investigate differences between groups of participants in age and handedness. For differences in sex, we used Fisher's exact test. Hemispheric asymmetries in patients were tested with the Wilcoxon test. We corrected with false discovery rate (FDR) for multiple comparisons. To check for correlations between changes in EEG and the severity of the motor deficits, as evaluated by NIHSS and WMFT scores, we assessed normality of data with Shapiro-Wilk tests and determined Pearson coefficients.

## 3. Results

The demographic characteristics of the stroke patients who were included in our sample did not differ significantly from those pertaining to the healthy participants, concerning age (*U* = 67.000, *p* = 0.150), sex (*p* = 0.700), or handedness as assessed by an adapted Edinburgh Handedness Inventory questionnaire [29] (*U* = 80.000, *p* = 0.272).

As described in Materials and Methods, we measured the individual active motor threshold for both healthy subjects and patients. Patients showed no significant differences in aMT values on the unaffected hemisphere, when comparing with healthy participants (*U* = 70.500, *p* = 0.785), thereby showing that these hemispheres were matched and enabling a fair comparison of neurophysiological profiles.

Concerning changes in neurophysiology following the stroke event, we assessed alpha rhythm at rest and motor rhythms, namely, mu and beta bands, during motor planning and execution.

**Figure 2:**
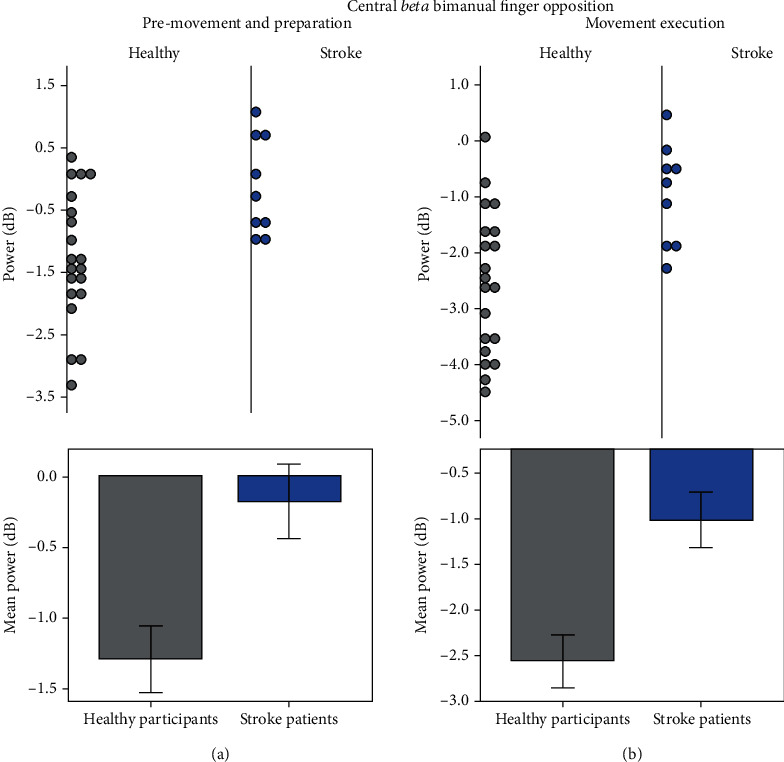
Beta power relative to baseline. Both groups showed desynchronization (negative mean power) with bimanual finger opposition. However, stroke patients did not increase beta desynchronization as much as the healthy controls. Significant differences (*p* < 0.05) are observed between healthy participants and stroke patients in power of the beta rhythm in the premovement and preparation and in the time-locked beginning of bimanual finger opposition. Error bars represent ±1 SE.

Even though both groups showed the expected beta desynchronization on the central motor areas (see Figure 1 for selection of electrode clusters) with simultaneous bimanual finger opposition, stroke patients showed significantly reduced ERD, in comparison with controls. This difference was significant both during premovement/preparation and on time-locked beginning of movement (*U* = 37.000, *p* = 0.011, Figure 2(a) and *U* = 31.000, *p* = 0.004, Figure 2(b), respectively).

**Figure 3:**
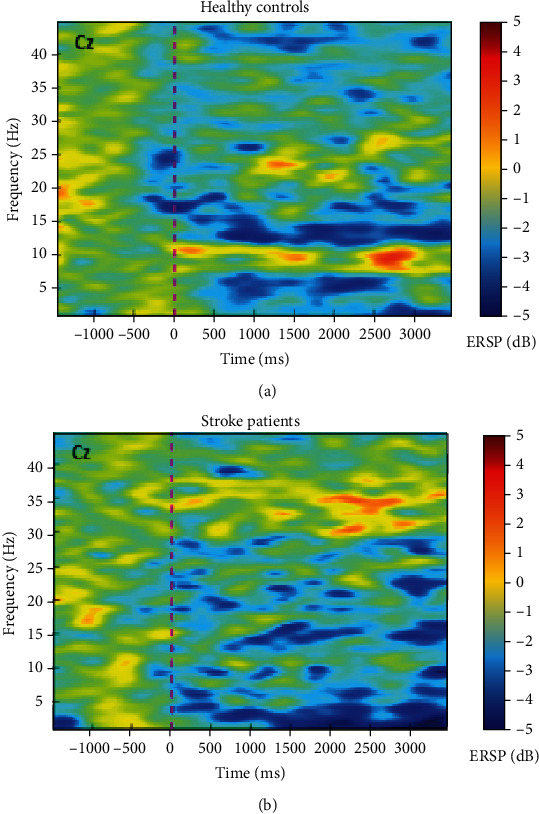
Group-averaged time-frequency plots for the motor area (central electrode, Cz), with bimanual thumb opposition task. (a) shows the time-frequency for healthy controls, while in (b), we present data from the stroke patients' group.

In Figure 3, we illustrate the group-averaged time-frequency plots, wherein we can distinctly observe the desynchronization pattern for the beta band in the motor area (Cz) of the control volunteers but not of the stroke patients.

**Figure 4:**
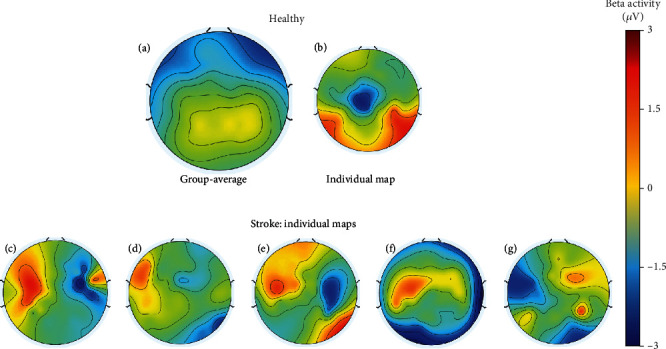
Grand-average topographical distribution for the beta rhythm of the control group (a), an example of an individual map from a healthy participant (b), and examples of individual maps for patients with a stroke lesion in the left (c–f) and right (g) hemispheres, during thumb opposition of both hands simultaneously. In each scalp map, *red* indicates synchronization, while *blue* is representing the desynchronization.

The differences in the beta band with the thumb opposition of both hands simultaneously coexisted with changes in the topography of individuals after a cerebrovascular lesion. In Figure 4, we compare stroke topographical distribution with that of a healthy brain, by presenting beta band scalp mapping during bimanual thumb opposition task.

Topographical distribution seems to corroborate the lower beta desynchronization (blue) in the central areas of stroke patients, comparing with controls. Moreover, in patients, the lesioned hemisphere showed a red pattern that suggests impaired modulation of beta oscillations.

Differences between healthy participants and stroke patients in alpha power of the posterior area were not significant, either when the subjects had the eyes opened (*U* = 68.000, *p* = 0.317) or closed (*U* = 72.000, *p* = 0.417). Mu rhythm did not show significant group differences when performing motor tasks with each upper limb (healthy or stroke-affected) individually or both simultaneously, either on shoulder flexion (*p* ≥ 0.183) or thumb opposition (*p* ≥ 0.077). Beta rhythm was not significantly altered in stroke patients comparing with healthy participants for shoulder flexion (*p* ≥ 0.216).

We found a significant moderate negative correlation between beta power during the execution of bimanual thumb opposition and the velocity of execution in WMFT tasks (r = −0.675, *p* = 0.046).

In patients, beta power in selected central electrodes (C3 and C4) did not show significant asymmetries between the affected and unaffected hemispheres on the preparation (*Z* = −0.652, *p* = 0.570) or execution (*Z* = −0.178, *p* = 0.910) of bimanual thumb opposition.

## 4. Discussion

The study of the hemisphere contralateral to the stroke lesion seems to be critical for the investigation of poststroke alterations [30]. The active motor threshold was assessed on the unaffected hemisphere, in patients, and randomly on the right or left hemisphere of healthy participants. After stroke, the hemisphere contralateral to the lesion is known to become overactive, which raises the hypothesis that the aMT in this hemisphere would be reduced. Our results however indicated only a nonsignificant trend for lower active motor threshold, suggesting that the hemisphere contralateral to the lesion was still relatively preserved. This is consistent with other findings. For example, Prashantha et al. analysed changes in the resting motor threshold of the nonaffected hemisphere compared with healthy controls and reported no differences at baseline (2 weeks after stroke onset), a trend for a decrease after 4 weeks of the lesion and a significant reduction on the second follow-up, at 6 weeks poststroke [30].

Our group-averaged time-frequency plots in the central Cz electrode revealed a distinct pattern of desynchronization with bimanual thumb opposition task in healthy subjects, which was not so evident in poststroke individuals. We found significant differences between patients and healthy participants in motor rhythms during thumb opposition, when performing the task with both hands simultaneously. These were observed as a lower reduction in beta power with the motor task, for patients, which indicates less desynchronization and suggests a less disinhibited state on central motor areas of stroke patients, when comparing to healthy subjects. Moreover, from the observation of topographical distribution in patients, we hypothesize that the impaired modulation of beta oscillations during movements including the affected hand might be detrimental to motor control. Bönstrup et al. [31] and Rossiter et al. [10] both assessed brain oscillations with paretic hand grip tasks in stroke patients, the first in the acute and the latter in the chronic phase of the disease, and described less movement-related beta decrease. Interestingly, Rossiter et al. did not detect changes in baseline power levels, reporting significant differences between groups only when studying dynamic changes with the motor task [10]. We suggest that, in our study, poststroke changes in oscillatory activity during bimanual thumb opposition were not circumscribed to the areas located near the lesion, which is supported by our results showing no significant asymmetries between powers on the electrode located in affected versus unaffected hemispheres.

Bartur et al. [12] found a correlation between the magnitude of ERD in the high-mu and low-beta bands and the motor function of the paretic upper limb, evaluated by EMG and by Fugl-Meyer and Box and Block tests, with better motor performance being correlated with greater desynchronization in the lesioned hemisphere only. In our work, we studied the correlation between WMFT and beta rhythm during bimanual movements and observed that patients who had more severe deficits (with slower execution in WMFT tasks) showed a significant correlated decrease in beta desynchronization with bimanual thumb opposition, which is in line with those results reported by Bartur et al. for the lesioned hemisphere. Our significant moderate correlation suggests that future studies, with large sample sizes, should further explore the potential of beta levels as biomarkers for stroke recovery of motor deficits. Actually, oscillations in the beta band are especially responsive to motor parameters [32]. Interestingly, Fu et al. [33] studied shoulder-elbow movement of the affected limb and also reported a significant decrease poststroke in peak ERD% in the mu range (8-12 Hz), comparing with healthy participants.

Regarding the shoulder flexion task, we were not able to detect significant differences between groups. This is consistent with the notion that movement complexity can influence the brain activation of the lesioned primary motor cortex [34]. Gerloff et al. [35] had already suggested that the involvement of M1 might be superior in more complex movement sequences, where there is larger activation of cortical areas. Puh et al. [36] pointed out finger movements as being the most suitable instruction when the focus is motor rehabilitation. The higher complexity involved in thumb opposition, associated to the motor control required for the transitions between fingers [36], can possibly explain the specificity of our results. This also provides insights into task dependence when probing neurophysiological changes in stroke and on the design of neurostimulation approaches.

This study has some limitations. Although it is crucial to analyse the neurophysiology of stroke in the acute and subacute stages, we cannot disregard the possibility that the timing of our experiment was too early to detect significant changes in the active motor threshold. Also, the effort required from poststroke patients to perform the motor tasks during electroencephalographic recording prevented us from including a larger number of trials for each movement. Despite this, we were able to find significant differences in motor rhythms, particularly in the beta band, in patients, when comparing with healthy controls.

The findings from this proof-of-concept study point out the value of studying EEG oscillations as potential biomarkers for understanding the neurophysiology of subacute stroke and the importance of conducting future work, with larger sample sizes, for potential application in clinical monitoring and novel therapeutic approaches.

## 5. Conclusions

We found that cerebrovascular lesions induced by recent ischemic stroke alter neurophysiological motor response patterns in both hemispheres translating into an alteration in event-related synchronization and desynchronization, particularly at beta frequencies during motor planning and execution of complex bimanual movements. These results have implications for tailoring neurostimulation strategies.

## Figures and Tables

**Table 1:** Demographic data of volunteers.

	Healthy participants *N* = 20	Stroke patients *N* = 10
Age (years; mean ± SD)	60.20 ± 11.237	67.10 ± 13.470
Sex (female/male)	11/9	4/6
Handedness (right/left-handed)	20/0	10/0

**Table 2:** Clinical data of stroke patients^†^.

Time since stroke (days; mean ± SD)	8.50 ± 1.581
Lesion side (right/left hemisphere)	4/6
NIHSS (mean ± SD)	6.40 ± 3.718
WMFT log performance time (mean ± SD)	2.14 ± 0.651
WMFT FAS (points; mean ± SD)	48.80 ± 31.255

^†^Abbreviations: FAS: functional ability scale; NIHSS: National Institutes of Health Stroke Scale; WMFT: Wolf Motor Function Test. The severity of the poststroke impairment increases with higher NIHSS scores, wherein a score of 0 would indicate no overall deficits and higher scores would represent greater deterioration of tested functions. WMFT scores (test described below) reflect the motor functionality of the affected upper limb. Lower performance time and higher scores in FAS are both associated with better performance.

## Data Availability

The data used to support the findings of this study are available from the corresponding author upon request.
